# Accuracy of pulse oximetry in detection of oxygen saturation in patients admitted to the intensive care unit of heart surgery: comparison of finger, toe, forehead and earlobe probes

**DOI:** 10.1186/s12912-018-0283-1

**Published:** 2018-04-17

**Authors:** Sohila Seifi, Alireza Khatony, Gholamreza Moradi, Alireza Abdi, Farid Najafi

**Affiliations:** 1 Students Research Committee, Kermanshah University of Medical Sciences, Kermanshah, Iran; 2 Nursing department, Nursing and Midwifery School, Kermanshah University of Medical Sciences, Kermanshah, Iran; 3 Department of anesthesiology, Medicine School, Kermanshah University of Medical Sciences, Kermanshah, Iran; 4 Research Center for Environmental Determinants of Health (RCEDH), School of Public Health, Kermanshah University of Medical Sciences, Kermanshah, Iran

**Keywords:** Pulse oximetry, Critical care, Accuracy

## Abstract

**Background:**

Heart surgery patients are more at risk of poor peripheral perfusion, and peripheral capillary oxygen saturation (SpO2) measurement is regular care for continuous analysis of blood oxygen saturation in these patients. With regard to controversial studies on accuracy of the current pulse oximetry probes and lack of data related to patients undergoing heart surgery, the present study was conducted to determine accuracy of pulse oximetry probes of finger, toe, forehead and earlobe in detection of oxygen saturation in patients admitted to intensive care units for coronary artery bypass surgery.

**Methods:**

In this clinical trial, 67 patients were recruited based on convenience sampling method among those admitted to intensive care units for coronary artery bypass surgery. The SpO2 value was measured using finger, toe, forehead and earlobe probes and then compared with the standard value of arterial oxygen saturation (SaO2). Data were entered into STATA-11 software and analyzed using descriptive, inferential and Bland-Altman statistical analyses.

**Results:**

Highest and lowest correlational mean values of SpO2 and SaO2 were related to finger and earlobe probes, respectively. The highest and lowest agreement of SpO2 and SaO2 were related to forehead and earlobe probes.

**Conclusion:**

The SpO2 of earlobe probes due to lesser mean difference, more limited confidence level and higher agreement ration with SaO2 resulted by arterial blood gas (ABG) analysis had higher accuracy. Thus, it is suggested to use earlobe probes in patients admitted to the intensive care unit for coronary artery bypass surgery.

**Trial registration:**

Registration of this trial protocol has been approved in Iranian Registry of Clinical Trials at 2018–03-19 with reference IRCT20100913004736N22. “Retrospectively registered.”

## Background

Pulse oximetry is a simple and non-invasive method used to examine oxygen saturation (SpO2) in various parts of body [1]. Using pulse oximetry is effective in accelerating the weaning from mechanical ventilation and extubation and reduces the frequency of bleeding for analysis of arterial blood gases (ABG), because for the patients who just need checking for the O2 saturation, pulse oximetry could be a proper alternative [2, 3]. Convenient use, speed and high accuracy in detection of hypoxia and continuous monitoring of patients are other features of pulse oximetry [3–5]. This device detects the amount of oxyhemoglobin and deoxygenated hemoglobin in arterial blood and shows it as Oxyhemoglobin saturation (SpO2) [6] which is an indirect estimation of arterial oxygen saturation (SaO2) [7]. The normal amount of SpO2 in healthy individuals is 97% to 99% [8].

If the SaO2 is 70% to 100%, the amount of SpO2 has high accuracy and is 2% different from the SaO2 amount obtained from ABG analysis [5]. Yet, in more critically ill patients, the amount of pulse oximetry error is reported as 7.2% [9]. Various factors can affect the accuracy of the device including the physiologic, environmental, technology failures and human error [1, 3, 7, 10–12].

There are contradictory and controversial results regarding the accurate detection of SpO2 by pulse oximeter obtained from the related studies [13–16]. Nessler et al. (2012) in their study concluded that among the patients under vasopressors, the forehead pulse oximeter sensor had higher accuracy in detection of SpO2 compared to transitional pulse oximetry of fingers [13]. The study of Bilan et al. (2006) indicated pulse oximetry by earlobe probe, had higher accuracy compared to pulse oximetry of finger and toe probes in detection of hypoxemia in children and babies. Further, it was shown that pulse oximetry of finger probes had the lowest agreement with SpO2 [14]. Korhan et al. (2011) suggested that in patients under physical restraints, the unfolded finger should be used to show the accurate value of SpO2 [15]. Wilson et al. (2010), in a retrospective cohort study, reported the difference of 2.7% between SpO2 and SaO2 in emergency patients with severe sepsis and septic shock and suggested using ABG where there is a need for more accurate detection of SaO2 [1]; the authors suggested doing more investigations due to the limitations of the study such as insufficient sample size.

Sugino et al. (2004) compared the pulse oximetry of forehead and finger probes in patients under general anesthesia. For this purpose, eighteen patients were induced by Propofol and time of lowest, time to recovery and lag time of beginning of SpO2 were measured for finger and forehead probes. The results showed that there are no differences between pulse oximetry of forehead and finger in terms of the mentioned times in a general anesthesia, and the authors suggested, the forehead probe can be a proper replacement when it is not possible to use finger probe [16]. Common methods such as forehead and finger probes have higher reliability in detection of peripheral oxygen saturation in patients with normal condition. However, these methods are not effective in critically ill patients hospitalized in intensive care unit with changes in vital signs because they have some limitations such as having edema in attached sites, and difficulties to matched control group [17, 18].

Considering the limitations and advantages of pulse oximetry in various parts of the body, the importance of accurate detection of hypoxemia and lack of studies about the proper method of pulse oximetry in patients admitted to intensive care units for coronary artery bypass surgery, the present study was conducted to determine the accuracy of pulse oximetry probes of finger, toe, forehead and earlobe in detection of oxygen saturation in the patients admitted to the intensive care unit for coronary artery bypass surgery.

## Methods

In this clinical trial, the study population was the patients admitted to the intensive care unit of Imam Ali (AS) Hospital affiliated to Kermanshah University of Medical Sciences (KUMS) for coronary artery bypass surgery. Study sample included 67 patients estimated based on the mean difference of 0.15 between measured SpO2 of finger, toe and forehead probes in the study by Yunt et al. (2011) [12], test power of 90% and probability error of first type as 5%.

These patients were selected based on convenience sampling and the inclusion criteria included having arterial line, oral temperature above 35 °C, Hemoglobin greater than 9 g/dl, mean arterial pressure of higher than 60 mmHg, PaO2 between 70% to 100% and pCO_2_ less than 45 mmHg, lack of underlying problems such as blood disorders (for example anemia, methemoglobinemia, carboxyhemoglobinemia), left ventricular failure, peripheral vascular disease, and acute and chronic renal failure, not having nail polish and finger clubbing, no history of smoking, and lack of ulcers, burns, edema and dressing in probe placement. Patients whose mean arterial pressure reached less than 60 mmHg or needed suction, received medicines affecting vessel diameter and had change position, were excluded from the study.

### Instrument

The portable probes of finger, toe and the forehead reflectance and earlobe pulse oximeter of Novametrx, Max-Fast, Nellcor Puritan Bennett INC, Pleasanton, Calif made in USA were used regarding all the patients for measurement of SpO2 values, respectively. In addition, four similar portable monitoring OXYPLETH 520A devices made in USA were used. The ABG reader XHOP SPLUL device made in USA was used in order to measure the ABG. Tympanic thermometer Jinus (series stat profile PHOX) was also used for measuring the temperature. In order to determine the reliability of ABG device which is considered as the standard of the study, two sample of arterial blood of 2 cm^3^ were taken from one of the patients. One of the samples was sent to the laboratory and the second sample was put in refrigerator after bleeding. The second sample was send to the laboratory in a time interval of two minutes following the first sample. Results of the study showed that there was an error of 0.11 between the SaO2 of both samples which showed the high reliability of the device.

Three blood samples of 2 cm^3^ were taken from one of the patients in order to determine the reliability of the ABG device. One of the samples was analyzed using the ABG reader device of the study and the other two samples were analyzed using another ABG reader device. Results of the study showed that there was a correlation of 0.93 between the SaO2 obtained from three ABG reader devices which indicated high correlation and reliability of the device. Four monitoring devices were of a same type and calibrated before the study. Tympanic thermometer was also calibrated prior to be used.

### Data collection

In order to collect the data, permission to conduct the study was issued by the Ethical Committee of KUMS. Then the required permissions were taken from Deputy of Research and Technology of KUMS and provided to the officials of the Imam Ali Hospital. Then, the researcher referred to the intensive care unit of the hospital every day and invited the patients having inclusion criteria. For this purpose, first of all the research purpose was explained to the patients and if they wished to be included in the study, they were asked to sign a written informed consent. The patients were assured about the anonymity and confidentiality of personal information. First of all, a blood sample 2 cm^3^ was taken from each patients through artery catheter by the researcher. Then the samples were put inside an ice container and immediately sent to the laboratory next to the ICU. Hb (Hemoglobin) and temperature of the patients were also recorded in the ABG test. Tympanic thermometer was used in order to measure the patients’ temperature. Further, the ratio and duration of SpO2 was also measured at the same time using the finger, toe, earlobe and forehead probes. It should also be mentioned that the same probes were used for all the patients.

Devices were calibrated prior to using the pulse oximetry. All the patients were in supine position and while the bed was 30 degrees above body surface area. A cover was put around the probes in order to prevent the intervention of environmental light with the performance of each four pulse oximetry probes. Moreover, unnecessary actions such as changing the position, suction, and medication were avoided while using probes to prevent any change in hemodynamic condition of the patients. SpO2 value showed on the monitor in finger, toe, earlobe and forehead probes were measured and recorded at the same time per 60 s for 5 min in time intervals of 0, 1,2,3,4 and 5 min. Finally, the mean SpO2 was calculated for each probe. The SpO2 values of each probe and also the SaO2 values were recorded on the information sheet.

### Data analysis

Data were analyzed using STATA-11 software. Independent t-test, Pearson correlation coefficient and Rho coefficient were used to compare the SpO2 and SaO2 of four probes. Bland- Altman analysis was used to compare the accuracy of each pulse oximetry probes. Lesser mean difference and higher agreement indicated higher accuracy of the probe. Kappa coefficient was used in order to divide the agreement ratio with the range of 0–1. Closer amounts indicate higher agreement [19].

## Results

Of the 67 patients, 56.7% (*n* = 38) were female and 43.3% (*n* = 29) were male. The mean and standard deviation of patients’ age were 57.22 ± 13.71 years. The mean and standard deviation of sample Hb was estimated as 13.21 ± 2.01 g/dl. The mean PaO2 in 43.3% of the sample was about 70–70.9 mmHg. The mean and standard deviation of the samples’ temperature were and 36.8 ± 0.6 °C and 58.2% of the sample had the temperature of 36–36.9 °C. The mean and standard deviation of PaCO2 were 35.03 ± 5.57 mmHg and this values for PaO2 were 96.81 ± 1.20%.

Based on the Pearson correlation test, the highest and lowest correlation between the mean SpO2 and mean SaO2 were related to earlobe probe (*r* = 0.77, *P*< 0.001) and toe probe (*r* = 0.60, *P*< 0.001). The highest and lowest clinical agreement with standard SaO2 were related to earlobe probes (0.76) and forehead probes (0.50), respectively. The confidence level (CI) calculated for clinical agreement in earlobe probe was less than other probes (Table 1).

**Table 1:** Comparing the correlation and agreement of SpO2 of four probes with standardized SaO2

Statistical index	Correlation			Agreement	
Probe type	r	*p*	CI	Rho	CI 95%
Finger	0.76	< 0.001	−1.02-2.08	0.68	−0.80- 0.57
Toe	0.60	< 0.001	−1.69- 2.28	0.58	−0.74- 0.43
Earlobe	0.77	< 0.001	−1.54- 1.83	0.76	−0.87- 0.67
Forehead	0.73	< 0.001	−1.07- 3.58	0.50	−0.62- 0.38

**Fig. 1:**
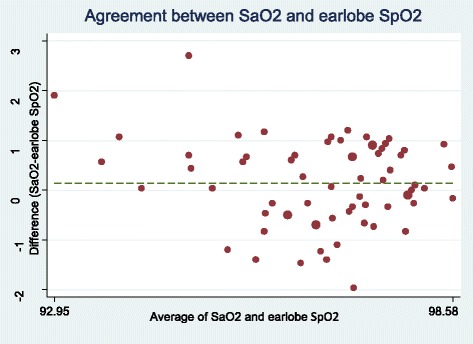
Bland-Altman plot of earlobe for measuring SpO2

**Fig. 2:**
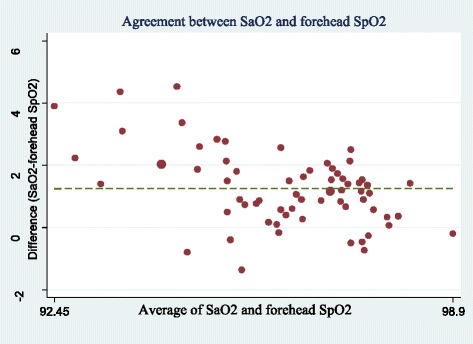
Bland-Altman plot of forehead for measuring SpO2

**Fig. 3:**
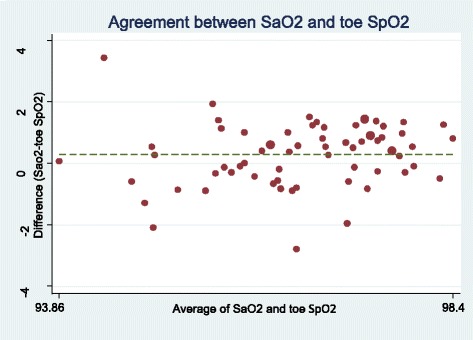
Bland-Altman plot of toe for measuring SpO2

**Fig. 4:**
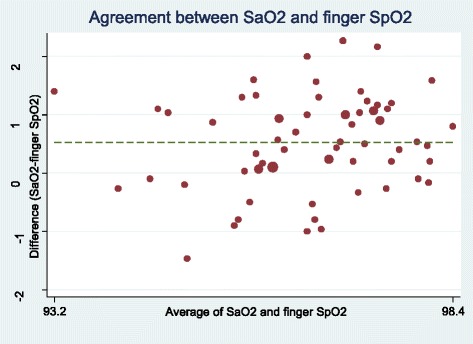
Bland-Altman plot of finger for measuring SpO2

The lowest mean difference of SpO2 or SaO2 was related to earlobe probe (0.14 ± 0.86) and the highest difference ratio was related to forehead probe (1.25 ± 1.18) (Table 2). Figures 1, 2, 3 and 4 show the Bland-Altman plot of comparing the clinical agreement between SaO2 or SpO2 values of earlobe, toe, forehead and finger probes where the earlobe probe with the agreement of 0.76 and confidence interval of 1.54–1.83 had the highest agreement with SaO2.

**Table 2:** agreement and mean difference of Finger, Toe, Earlobe and Forehead pulse oximeters comparing to Standard SaO2

Statistical index	Mean	SD	Mean difference SaO2-SpO2	*p*-Value for t-test of mean difference	CI 95% for agreement
SpO2 probe					
Forehead	95.55	1.75	1.25 ± 1.18	< 0.001	0.38–0.62
Earlobe	96.67	1.34	0.14 ± 0.86	0.019	0.67–0.87
Finger	96.28	1.06	0.53 ± 0.79	< 0.001	0.57–0.80
Toe	96.52	1.06	0.29 ± 1.01	0.22	0.43–0.74

## Discussion

In this study, the earlobe probes had the highest clinical agreement with SaO2 and higher accuracy due to less mean difference and limited confidence interval following by the finger, toe and forehead probes. Results of the study by Bilan et al. (2010) indicated pulse oximetry of earlobe probe had higher accuracy compared to pulse oximetry of finger and toe probes in detection of hypoxemia in children and babies. Further, it was shown that pulse oximetry of finger probe had the lowest agreement with SaO2 [4]. However, Vax et al. (2009) compared the ear and forehead probes in patients under coronary surgery and reported that forehead probe had higher clinical agreement compared to ear probe [20]. Benz et al. (1991) also examined the accuracy of Biox 3700 pulse oximeter in patients receiving medicines affecting vessel diameter and reported that the ear probe of patients receiving medicines affecting vessel diameter was not reliable for monitoring SaO2 [21]. It seems that measuring the oxygen saturation using pulse oximeter can be influenced by the patient’s condition; in this regard, Eberhard et al. (2002) stated that earlobe probe was the proper method in patients under anesthesia due to high speed of detecting SaO2 [22]. Lindholm et al. (2007) also argued that earlobe probe was the more proper method compared to finger probe due to more sensitivity to peripheral hypoxia in detection of SaO2 in patients with apnea [23]. Simon et al. (2003) also showed that ear probe was a more proper approach compared to finger probe in hypothermia [24].

However, other studies had different results based on patients’ condition [7, 25–27]. In this regard, Fernandez et al. (2007), in a method-comparison study, assessed the agreement between SpO2 values by digit-based and forehead pulse oximeters in low cardiac index patients, they suggested using forehead probe in these patients since the peripheral blood flow and body temperature are decreased [25]. Schallom et al. also claimed that in critically ill patients under surgery or with trauma exposed to the risk of peripheral hypoperfusion, the SpO2 ratio by forehead probe was more accurate than finger probe [26]. Das et al. (2010) also believe that there is no proper method for the placement of pulse oximeter sensor in children with cyanotic heart disease due to their specific condition to show the accurate value of SpO2. However, foot sensor can be the more appropriate one in this regard [7]. Sedaghat Yazdi et al. (2008) examined the effect of placement on the accuracy and reliability of the pulse oximeter sensor in children with cyanotic heart disease and found that if the SaO2 ≤ 90%, the foot sensor had the worst accuracy and reliability. Further, regardless of SaO2 amount, there was no difference between finger and toe sensor in terms of accuracy and reliability [27].

Although the accuracy of pulse oximetry is reduced in patients with severe and rapid O_2_ desaturation, low blood pressure, body temperature, dyshemoglobinemia and reduced blood perfusion conditions [28], the earlobe pulse oximetry had more accurate and reliable performance regarding these changes [22, 23, 29]. Haynes (2007) claimed that earlobe probe can be considered as the proper method of finger pulse oximetry since in finger probe, the body movement is limited and the risk of reduced tissue perfusion is increased [18]. Thus, regarding the importance of continuous monitoring and maintaining hemodynamic stability in patients under heart surgery [29] and considering the results of our study, the earlobe probe can be used as the proper method for examining the oxygen saturation in patients under heart surgery.

In this study, the environmental light could intervene with the performance of each four pulse oximetry probes. However, a cover was put around the probes in order to prevent the intervention of environmental light with the performance of each four pulse oximetry probes. Non-random sampling was used for this study which could affect the generalizability of the findings. Thus, it is suggested to replicate the study using random sampling in various patients. The study was conducted on the patients admitted to the intensive care unit for cardiac surgery and it is suggested to conduct the similar study on the patients admitted to emergency and operating rooms.

## Conclusion

Results of the study indicated that earlobe probe had higher accuracy in showing the SpO_2_ among patients admitted to the intensive care unit for heart surgery compared to finger, toe and forehead probes and the obtained SpO_2_ value of earlobe probe approximated to the SaO_2_ obtained from ABG test. Thus, earlobe probe can be used in intensive care units to measure the peripheral oxygen saturation.

## Abbreviations

ABG: Arterial Blood Gases
C: Centigrade
Hg: hemoglobin
KUMS: Kermanshah University of Medical Sciences
Sao2: Saturation of Oxygen (arterial blood)
Spo2: Spot Oxygen Saturation
